# Spherical Boundary Conditions: A Topological Framework for Isotropic Collective Dynamics

**DOI:** 10.1021/acs.jpcb.6c01770

**Published:** 2026-05-28

**Authors:** Manuel Dedola, Ludovico Cademartiri

**Affiliations:** Department of Chemistry, Life Sciences and Environmental Sustainability, 9370 University of Parma, Parco Area delle Scienze 17 A, Parma 43121, Italy

## Abstract

Standard periodic boundary conditions (PBC) impose a toroidal topology on simulation domains. While geometrically flat, this topology allows for coherent reentrant self-interactions that preserve spurious long-range temporal correlations. We show that these topological artifacts manifest as strong, lattice-aligned anisotropy in collective dynamic observables, rendering scalar relaxation rates direction-dependent even at near-particle length scales, thereby violating the static–dynamic correspondence of isotropic liquids (de Gennes narrowing). To resolve this, we introduce Spherical Boundary Conditions (SBC), a topological framework that replaces the periodic torus with a mixing quotient space. SBC is defined by a radial folding map coupled to a boundary remapping driven by deterministic chaos. This construction effectively acts as a measure-preserving information filter: it preserves thermodynamic conservation laws, while suppressing the Lagrangian memory responsible for periodic artifacts. Using Brownian dynamics simulations, we demonstrate that SBC eliminates lattice-aligned anisotropy by construction, recovering isotropic static and dynamic correlations and effectively restoring the ergodicity and static–dynamic correspondence of the infinite bulk limit on a finite support.

## Graphical abstract

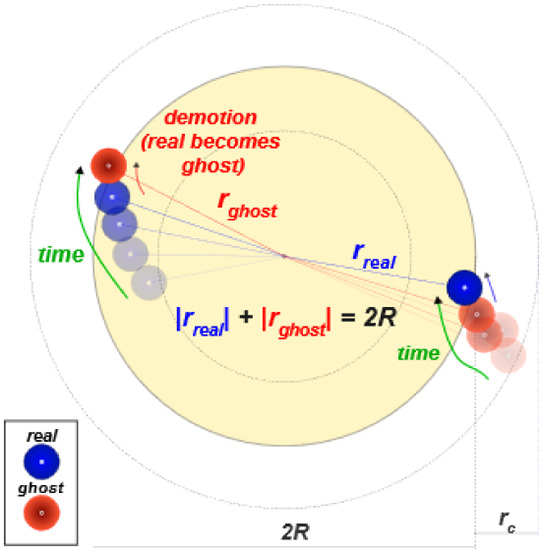

## Introduction

Collective dynamics in liquids mediate the coupling between microscopic particle motion and macroscopic transport phenomena.^1,2^ The influence of these collective modes is not necessarily limited to hydrodynamic length scales. For example, in crowded, overdamped systems, local structural evolution is governed by relaxation time scales and spatial coherence that are controlled by the collective transport coefficients.^3,4^ Extensive prior work has demonstrated how dense, crowded environments significantly modify transport, often leading to anomalous diffusion.^5–7^ These collective dynamics are quantified by wavevector-dependent observables such as the intermediate scattering function *F*(***k***,*t*) and the collective diffusivity *D*_eff_(***k***). The latter is defined as $D_{eff}\left(k\right)=\frac{\Gamma \left(k\right)}{k^{2}}$ , where Γ­(***k***) represents the initial decay rate of $F\left(k,t\right)∼e^{-\Gamma \left(k\right)t}$ , and it quantifies the relaxation of density fluctuations at a specific length scale. Accurately capturing observables like *D*_eff_(***k***) without artifacts is especially critical when studying the slowing down of diffusion-controlled reactions and equilibria in such crowded media.^8,9^

Simulating these bulk properties in finite volumes requires boundary conditions. The standard approach—Periodic Boundary Conditions (PBC)—prioritizes the elimination of surface effects by tiling a space-filling domain. This topological closure introduces distinct artifacts arising from both the shape of the observation domain (convolution with a geometric form factor) and the discrete translational symmetry of the lattice (cf. Figure 1).^10–15^

**Figure 1:**
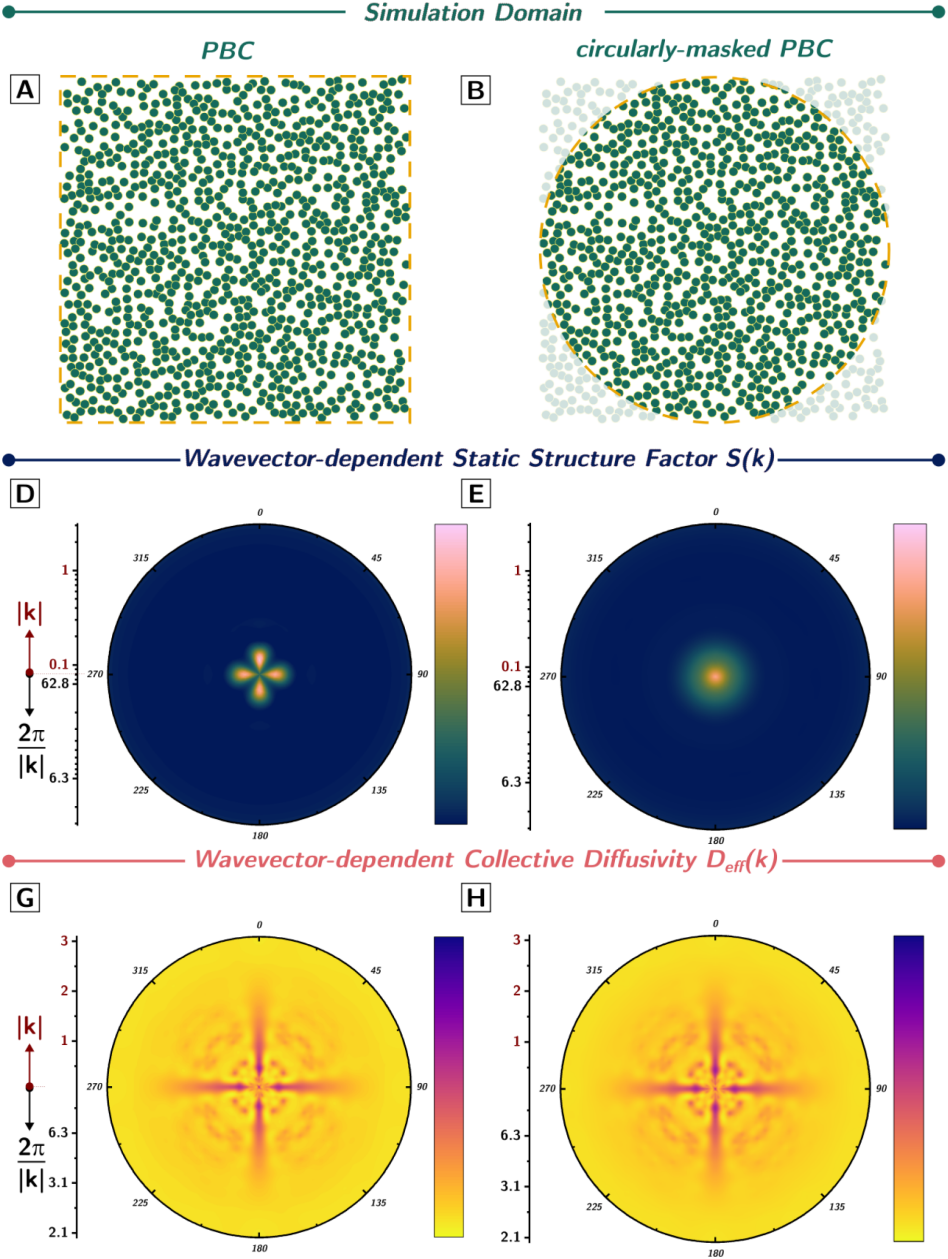
Visualization of boundary shape and periodicity effects on statics and dynamics. The first column (panels A, D, G) shows results from a 2D Brownian simulation in PBC where the entire square observation domain (A) was considered in the characterization of the static structure factor S­(k) (D) and collective diffusivity *D*_eff_(***k***) (G). The second column (panels B, E, H) instead shows the same results when the only area considered for characterization of *S*(***k***) (E) and *D*_eff_(***k***) (H) was the inscribed circle (B). The data show how the anisotropy in the *S*(**k**) in PBC is due to the polyhedral shape of the observation window and can therefore be isotropized by truncating to an inscribed circle. The same is not true for *D*_eff_(***k***), where anisotropy is caused by translational periodicity.

These artifacts affect static and collective dynamic properties differently. While geometric masking of the simulation domain (e.g., analyzing an inscribed sphere^16^) can recover apparent isotropy in static observables like the static structure factor *S*(***k***), it fails to correct collective dynamic observables like *D*_eff_(***k***) (cf. Figure 1).^17–19^ Static observables are calculated from instantaneous domain configurations: they encode the shape of the domain, but are oblivious to what lies outside it (e.g., periodic equivalents). Dynamic observables describe instead the evolution of configurations flowing through the domain: they encode the shape of the domain, but also detect its translational periodicity.^20–22^

These known trade-offs of PBCs are accepted due to their astounding success in reproducing scalar thermodynamic quantities and local geometries.^23^ Yet, an alternative ensemble that makes a different trade-off—i.e., relaxing periodicity, while preserving a homogeneous interaction environment—could be valuable for the study of collective transport phenomena.^24–27^ Such departures from periodicity typically result in systems that are either open^28–30^ or bounded by hard walls,^31,32^ both of which have known challenges.^32,33^ Here, we introduce a boundary condition that is neither open nor confined.

## Theory and Implementation of Spherical Boundary Conditions

Our ensemble is defined within a sphere of diameter 2*R* (cf. Figure 2A). Every particle whose center of mass (COM) lies inside the domain (“real”) has a counterpart (“ghost”) outside the domain. Ghosts are “tethered” to their real counterpart only by a holonomic radial mapping (“tether rule”: | ***r***_ghost_| = 2*R* – | ***r***_real_| where 2R is the “tether length”). Their angular coordinates are instead unconstrained and evolve according to the same equations of motion as the real particles. Since the tether length is equal to the domain diameter, if a real particle’s COM exits the domain, the COM of its ghost necessarily enters, leading to the handover: the ghost is promoted to real and the real demoted to ghost.

The algorithmic simplicity of the “tether rule” betrays the nontriviality of its consequences:

**Thermodynamically**, the SBC simulation domain is a closed system despite the external “ghost” layer: the radial handover mechanism enforces a strict conservation of particle number *N* and therefore the partition function of the system is Canonical. Mathematically, SBC implements a zero-flux (Neumann) boundary condition on the particle density current, distinct from the Dirichlet or open boundary conditions associated with Grand Canonical reservoirs.^34^ SBCs therefore create a Canonical (NVT) ensemble embedded within a stochastic, radially coupled mean field generated by the dynamically evolving ghost population.^28–36^

**Topologically**, SBCs operate as a conservative, topological, mixing engine. Unlike PBCs, which connect space in an information-conserving loop, the SBC boundary is a topological scrambler. When a particle reaches the edge, it is exchanged with a partner from a dynamically evolving auxiliary phase space. This operation shreds the spatial correlations and Lagrangian memory of the trajectory, preventing the re-entrant artifacts typical of small simulation boxes. This scrambling is achieved purely through topology: particle positions are mixed without adding artificial friction, noise, or energy. Below is a more technical explanation of the subtleties of SBCs in terms of (i) geometric topology of phase/configuration space, (ii) deterministic chaos and ergodic theory, (iii) equilibrium statistical mechanics, and (iv) symplectic geometry.
  A rigorous way to describe SBC’s topology is as a fiber bundle structure. Let the configuration space be decomposed into a bundle, where the base manifold B represents the radial coordinate (*r* ∈ [0, *R*]), and the fiber F represents the angular manifold (*S* ^2^). Standard PBCs construct a torus *T* ^3^ by imposing a static identity map between the fibers at opposite boundaries of a Cartesian base. In contrast, SBC is defined by a folding map on B (the tether rule) coupled with deterministic chaotic automorphism on the fibers (or, more rigorously, a chaotic, time-dependent, state-dependent remapping). Although the mapping is (by itself) fully deterministic and time-reversible (dependent on the instantaneous microstate of the ghosts), the intrinsic instability generated by the ghost dynamics compromises the specific nonergodic invariants associated with toroidal geometry (e.g., infinite loops).
  This distinction is fundamental: the SBC boundary acts as an effective Pseudorandom Number Generator (PRNG) realized by deterministic many-body dynamics. It provides the statistical benefits of a stochastic boundary (decorrelation), without injecting any boundary-specific stochastic forcing (beyond the Brownian dynamics already present in the bulk). The entropy associated with the boundary dynamics is Kolmogorov–Sinai entropy (i.e., intrinsic to the dynamics), not thermodynamic entropy created coupling to an heat bath. SBC differs from billiard-like reflections: the folding acts on configuration space rather than velocity space, producing trajectory separation using topology rather than energy injection: the handover event corresponds to a jump between distinct fibers in the angular configuration space. The SBC boundary creates a hyperbolic scatterer: when a bundle of arbitrarily close trajectories hits the boundary, they are mapped to trajectories that are macroscopic distances apart (effectively infinite local sensitivity to initial conditions). Importantly, while SBC enforces rapid decorrelation and effective ergodicity on a finite support, it does so through a mechanism compatible with the symplectic structure of Hamiltonian flow: the tether rule is a time-independent holonomic constraint acting solely on configuration space, and the handover event corresponds to a relabeling of degrees of freedom rather than a modification of momenta or forces. This is the profound difference between SBC and thermostats (it does not rescale velocities), stochastic walls (it does not draw new velocities from a distribution), and dissipative walls (it does not remove energy). While the simulations here employ Brownian dynamics, the SBC formalism itself is derived from time-reversible holonomic constraints. Unlike stochastic or dissipative walls, SBC would strictly preserve energy and phase space volume if applied to Newtonian (NVE) dynamics. Viewed from a higher level, SBC can be interpreted as a dynamical system in which the boundary condition is not a static geometric constraint, but a dynamic coupling to an auxiliary phase space. Importantly, this coupling is deterministic, time-reversal symmetric, and implemented through a holonomic constraint on configuration space, thereby capable of preserving the symplectic structure of the Hamiltonian flow.
  Topology-based alternatives to PBCs have been explored previously, most notably simulations performed on closed curved manifolds such as hyperspheres.^24,37–40^ These approaches eliminate explicit boundaries by global closure of space and preserve full Lagrangian continuity. As a consequence, while they remove Cartesian anisotropies and periodic images, they retain coherent reentrant trajectories and long-time self-correlations inherent to closed topologies. SBCs differ fundamentally in that they retain an open domain while destroying re-entrancy through identity exchange at the boundary, leading to qualitatively different consequences for collective relaxation dynamics.
**Kinematically**, the SBC is not a coordinate wrapping of a single particle, but an exchange mechanism between distinct populations that conserves mass. In other words, the number of particles stays constant, but their Lagrangian identities do not. When a particle *i* exits the real domain, it does not “teleport” algorithmically; rather, it transitions with kinematic continuity into the ghost domain, effectively leaving the observation window. Simultaneously, the tether rule ensures that a distinct particle *j* from the ghost domain crosses with kinematic continuity into the observation window. Unlike PBCs, where a particle carries its neighbor cage with it across the periodic face, the local environment of the entering particle *j* is statistically independent from that of the exiting *i* particle. Therefore, while thermodynamic quantities (*N*, *V*, *T*) are conserved, the Lagrangian trajectory of a specific particle index is reset upon boundary crossing, rendering long-time trajectory unwrapping ill-defined.
**Dynamically**, the exchange at the boundary truncates Lagrangian memory. In a standard closed system, a particle carries its “memory” (momentum, identity, and local history). In SBC, history is truncated at every boundary crossing. The disadvantage is that one cannot calculate any observable (e.g., Mean Squared Displacement, MSD) that requires tracking a single specific particle past a crossing. The advantage is that by forgetting the trajectory at the boundary one eliminates spurious reentrant self-correlation (e.g., a particle interacting with its own wake).
**Observationally**, collective dynamics must therefore be characterized through Eulerian density correlators such as the intermediate scattering function *F*(***k***,*t*), which remain well-defined under identity exchange.
**Computationally**, SBC requires explicit time integration only for ghost particles within a shell of thickness *r*_c_ near the boundary, causing the cost of the boundary condition to scale with surface area rather than volume. From a practical implementation standpoint, integrating SBC into existing molecular dynamics engines incurs minimal algorithmic complexity. The core mechanisms—the radial tether rule and the promotion/demotion handover—require only basic algebraic evaluations and conditional checks applied to particle coordinates at each integration step.

In the following, we present results from overdamped Brownian Dynamics (BD) simulations designed to (i) examine the behavior of the spherical boundary condition (SBC) introduced here, and (ii) compare it directly with conventional periodic boundary conditions (PBC). Simulations employ classical particles interacting via short-range pairwise potentials. The Lennard–Jones (LJ) potential serves as the primary interaction model. Three boundary conditions are considered: PBC in a cubic simulation cell, PBC implemented on a face-centered-cubic (FCC) lattice, and SBC.

**Figure 2:**
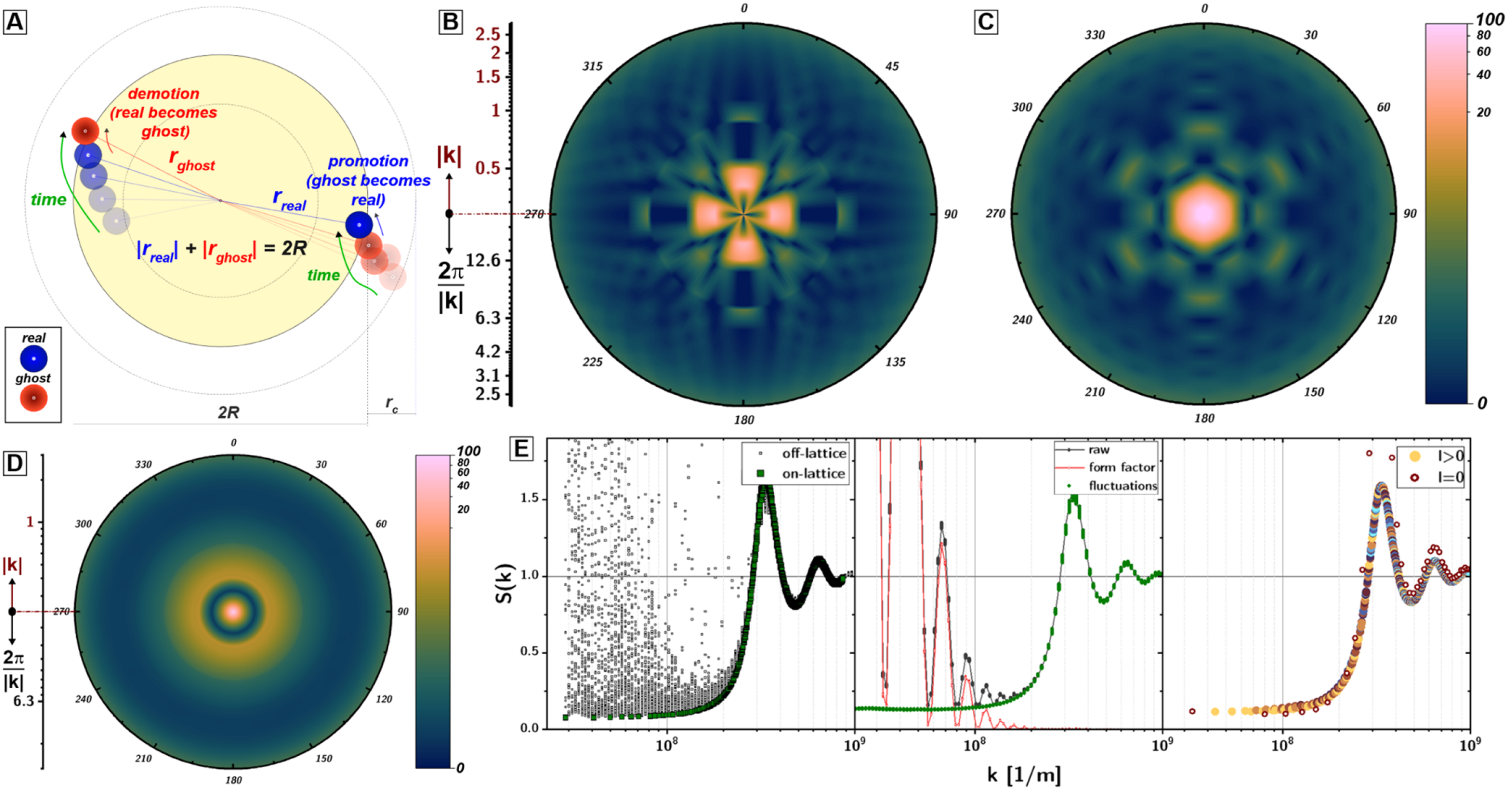
Static structure under periodic and spherical boundary conditions. (A) Schematic of the spherical boundary condition (SBC), illustrating the relation between real and ghost particles and the interaction halo of thickness *r*_c_. (B) Polar representation of the static structure factor *S*(***k***) obtained under cubic periodic boundary conditions (PBC), showing pronounced anisotropy aligned with lattice symmetry. (C) The same quantity obtained under FCC periodic boundary conditions, demonstrating reduced but persistent anisotropy. (D) log_10_(*S*(***k***)) obtained under SBC, showing isotropy without geometric masking. (E) One-dimensional diagnostics of static structure with shared *S*(***k***) ordinate axis: (left) spectral leakage in PBC for off-lattice wavevectors; (middle) separation of raw signal, spherical form factor, and fluctuation spectrum in SBC; (right) recovery of a smooth isotropic spectrum using a spherical Bessel basis, including isolation of the *l* = 0 mode. The colormaps are shown on a logarithmic scale to resolve angular modulations of *S*(***k***) and are perceptually uniform and ordered; labels in k and 2π/***k*** axes are multiplied by 10^8^ for clarity.

Collective structure and dynamics are characterized by a common set of observables across all ensembles: *S*(***k***), *D*_eff_(***k***), the intermediate scattering function *F*(***k***,*t*), the radial pair distribution function *g*(| ***r*** |), its angularly resolved counterpart within the nearest-neighbor shell, and distributions of waiting and residence times. Simulation parameters are identical across boundary conditions and are summarized in Table 1.

**Table 1:** Simulation Parameters

Parameter	Value	Parameter	Value	Parameter	Value
*N*	1000	**interaction potential**	LJ	**time step**	11.4 ns
volume fraction φ	0.4	**ε**	1 k_B_T	**number of timesteps**	10^6^
radius *r*_p_	10 nm	***T*** *, ρ*	1, 0.76	**total time simulated**	11.4 ms
phases	SiO_2_ in H_2_O	**σ**	2*r*_p_ + 0.25 nm hydration layer	**global relaxation time**	∼0.7 ms
subsampling correction α	0.7071	interaction cutoff, ***r***_**c**_	3σ	**step displacement/** **particle radius**	0.11

This work does not aim to propose SBC as a universal replacement for periodic boundary conditions, nor does it address long-range electrostatics, Ewald-type methods, or interfacial systems. Our focus is narrower: to identify alternative boundary conditions with complementary weaknesses to PBCs, especially in the context of anisotropy.

## Results

### Static Structure under Periodic and Spherical Boundary Conditions

Figure 2B-E examines how the angular dependence of static density correlations is shaped by domain geometry and basis choice. Under PBCs, the angular dependence of *S*(***k***) (Figure 2B-C) is dominated by the form factor of the finite simulation domain rather than by the intrinsic structure of the fluid. As shown in Figure 1, *S*(***k***) is insensitive to the periodic lattice *per se* but is convolved with the Fourier transform of the observation window.

Isotropic static structure *can* be extracted from PBC simulations by direct computation of the raw scattering signal on a continuous set of wavevectors using spherically masked real-space data (cf. Supporting Information, Figure S1). This procedure, while possible, is not without cost, as it requires discarding a substantial fraction of the configuration-space information (≈48% for cubic PBC and ≈60% for FCC PBC).

Under SBC, the simulation domain is spherically symmetric: its S­(**k**) (Figure 2D) is isotropic without the need for geometric masking or angular averaging. The continuous fluctuation spectrum can be losslessly obtained by projecting randomly oriented wavevectors onto the canonical plane-wave basis and removing the smooth spherical form factor which is known analytically (Figure 2E, middle). By contrast, using off-lattice vectors in PBC leads to the well-known problem of spectral leakage (Figure 2E, left).

If calculated on its native spherical Bessel representation, the raw SBC signal forms a smooth radial spectrum that allows explicit isolation of the *l* = 0 mode (Figure 2E, right), which captures any residual inhomogeneity associated with the spherically symmetric domain boundary.

### Collective Dynamic Transport under Periodic and Spherical Boundary Conditions

Figure 3 presents the angularly resolved collective diffusivity *D*_eff_(***k***) for the three boundary conditions, evaluated on a common absolute color scale and over the same range of wavevectors spanning the entire k-range (from 2π/*L* to π/*r*_p_). Panels A and B show results obtained under cubic and FCC PBCs, respectively, after spherical masking of the real-space data to remove trivial geometric asymmetries of the observation window. Panel C shows the corresponding results obtained under SBCs.

**Figure 3:**
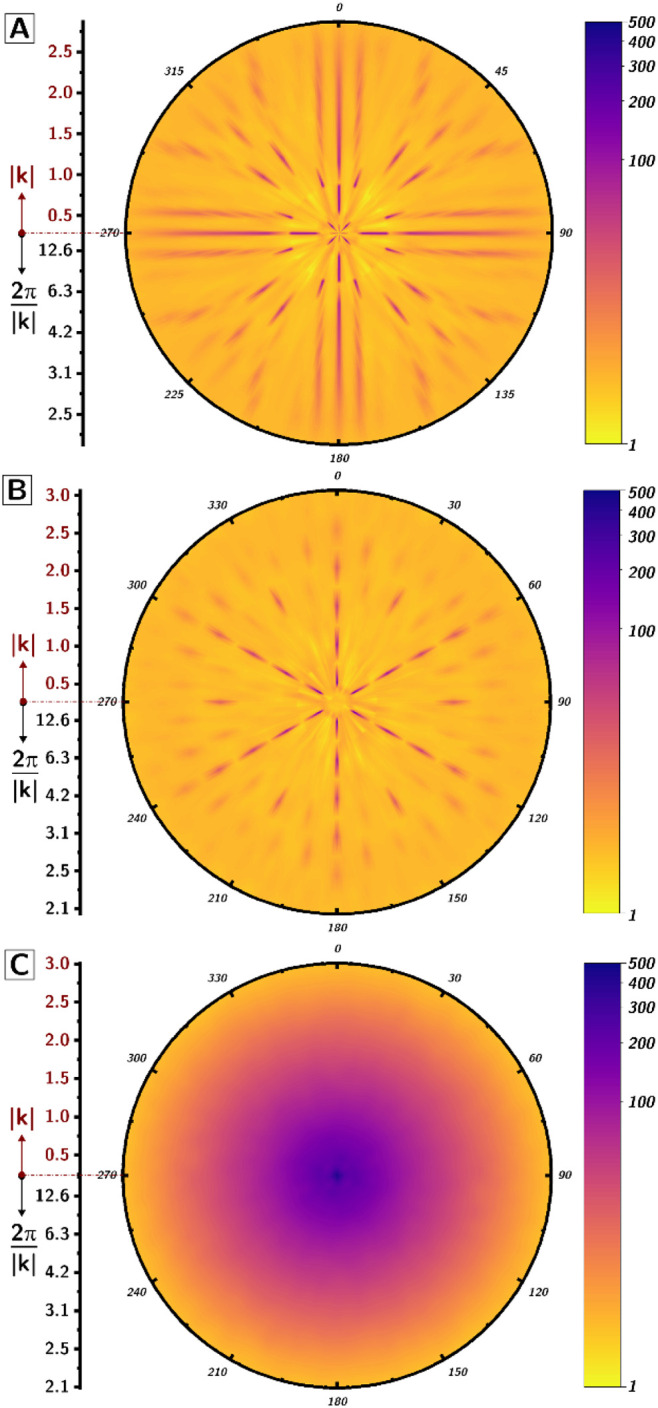
Dynamic transport under periodic and spherical boundary conditions. Angularly resolved collective diffusivity *D*_eff_(***k***) for different boundary conditions. Periodic boundary conditions implemented on a (A) cubic lattice and on a (B) FCC lattice, shown on a common absolute color scale. Strong angular anisotropy persists despite spherical masking of the data. (C) Spherical boundary conditions (SBC) shown on the same absolute scale, revealing a collapse of the angular dynamic range and the absence of lattice-imposed symmetry. Panels A and C are sections corresponding to the xy plane; panel B is a section perpendicular to the [111] direction. Axes labeled in | ***k*** | and 2π/| ***k*** | are multiplied by 10^8^ for clarity. All | ***k*** | axes span the range from 2π/*L*, where *L* is the domain size, to π/*r*_p_, where *r*_p_ is the particle radius. As indicated by the 2π/| ***k*** | (expressed in units of particle radii), angular anisotropy in *D*_eff_(***k***) under PBC extends into the nearest-neighbor shell at these volume fractions φ.

Despite masking to remove the influence of the domain’s form factor, strong angular structure persists in *D*_eff_(***k***) under both periodic ensembles. In the cubic case (Figure 3A), the anisotropy is aligned with the Cartesian axes of the simulation cell and extends continuously from the longest wavelengths down to wavevectors probing the nearest-neighbor shell. Implementing PBC on an isotropized FCC lattice (Figure 3B) does not eliminate the anisotropy: discrete symmetry directions remain clearly visible, the angular dynamic range remains large, and the anisotropy still persists at length scales of 1–2 particle diameters.

By contrast, *D*_eff_(***k***) obtained under SBC (Figure 3C) is isotropic within statistical resolution across the entire accessible range of wavevectors. The angular dynamic range is reduced compared to periodic ensembles, and no residual lattice-imposed symmetry is observed. Notably, this isotropy is achieved without geometric masking, angular averaging, or post hoc correction of the data.

### Time-Domain Anisotropy under Periodic and Spherical Boundary Conditions

While the polar topographies in Figure 3 visualize the structure of the anisotropy, Figure 4 quantifies its magnitude and its effect on the angularly integrated values of the dynamic observables.

The most information-rich dynamic observable is *F*(***k***,*t*), the decay in time *t* of the amplitude of the mode shape indicated by the wavevector ***k***. Before we discuss the *F*(***k***,*t*) results, it is worth reminding ourselves of two relevant and fundamental characteristics of the physics of isotropic liquids: (i) *F* should depend only on | ***k*** | and *t* (i.e., angular spread should be noise-limited), and (ii) collective fluctuations do not recur coherently.

We then picked two | ***k*** | values representative of two important length scales: an intermediate scale capturing semilocal dynamics | ***k*** | = 2π/3*r*_p_ and the system scale | ***k*** | = 2π/*L*. For each of these, we calculated the *F*(***k***,*t*) for ∼400 wavevector orientations (spherical Fibonacci point sets). Figure 4A shows the angular averages (solid lines) for both length scales and for all three boundary conditions. The overlaid shaded regions are instead the CI95 of the respective averages, i.e., the angular spread of the observable in the entire solid angle.

The boundary conditions have large differences in the dependence of *F*(***k***,*t*) on both ***k*** and *t*, at both length scales. This behavior is similar to what is observed for the SBC ensemble (yellow) at both length-scales: the shaded region is barely visible, confirming the results of Figure 2 that the relaxation dynamics show little rotational variation. Both cubic (teal) and FCC (purple) PBCs exhibit a much larger directional spread at both length-scales.

The decay rates are also very different. At both length scales, SBC angular averages decay much faster than PBC averages. The collective dynamics of the three boundary conditions is fundamentally different at both length scales. It is not just a difference in angular variance that can be dealt with by integration.

This difference is usefully visualized by plotting the decay rates Γ as a function of the azimuthal angle θ in the same length scale described above (cf. Figure 4C-D). At the intermediate scale (Figure 4C), where boundary effects could be expected to be negligible, the PBC data (cyan and purple curves) betrays the presence of the lattice. The relaxation rate is not constant as it ideally ought to be, but is instead characterized by sharp, periodic spikes where the effective diffusivity increases significantly along specific symmetry axes. The FCC is better only in that there are fewer peaks. In contrast, the SBC data (yellow) fluctuates randomly around a constant (and much higher) mean, consistent with a truly isotropic fluid.

In the hydrodynamic limit (cf. Figure 4D), the distinction is categorical (the ordinates are now plotted in log scale). The SBC signal remains isotropic. The cubic PBC retains its 4-fold anisotropy of sharp peaks separated by a baseline that is nearly 2 orders of magnitude lower than the average SBC value. The FCC signal appears flat but is overlapped with the baseline of the PBC: this is not isotropy, but a “spectral silence” caused by scanning a plane where the FCC reciprocal lattice has no allowed modes (a spectral gap). It is noteworthy that most peak values of the PBCs’ Γ are still lower than the plateau value of SBC.

Periodic boundaries enforce topological closure, which causes coherent recursion of collective fluctuations. Even for wavevectors aligned with lattice symmetry directions, collective modes remain confined and repeatedly reenter the observation volume with preserved phase coherence, resulting in slower relaxation (“ringing”).^17^ In the thermodynamic limit of an infinite, homogeneous fluid, such coherent recurrences are absent and collective fluctuations decay without echo.

The spherical folding employed in SBC leaves tangential directions topologically open. Collective fluctuations that leave the core region undergo irreversible phase mixing in the tangential degrees of freedom and do not return as coherent echoes. Consistent with this absence of mode recurrence, decay rates under SBC are systematically higher, including modes that would be optimally supported under PBC. The faster decay rates under SBC differ fundamentally from physical memory effects like crowding-induced renormalized diffusivity. While physical crowding alters diffusion through genuine local multiparticle caging, SBC’s effect is purely topological. It eliminates the spurious Lagrangian memory and coherent re-entrancy caused by periodic boundaries. As evidenced by our collision statistics, SBC does not alter underlying hydrodynamic correlations or local physical dynamics; it strictly removes the macroscopic topological artifacts that artificially prolong collective relaxation.

### Static–Dynamic Correspondence

In isotropic liquids, equilibrium structure and collective dynamics are linked by a well-established static–dynamic correspondence. In particular, the relaxation rate of density fluctuations at wavevector ***k*** is inversely related to *S*(***k***), a result commonly referred to as “de Gennes narrowing”. This relation follows from isotropy, linear response, and irreversible phase mixing of collective modes, and it implies that, at fixed | ***k*** |, collective relaxation rates are scalar quantities independent of direction.

Under PBCs, we observe a violation of this correspondence. While the static structure factor *S*(***k***) can be rendered approximately isotropic by geometric averaging over reciprocal lattice shells, the corresponding intermediate scattering function *F*(***k***,*t*) exhibits strong directional dependence. As a result, relaxation rates extracted from *F*(***k***,*t*) vary by orders of magnitude for wavevectors of identical magnitude but different lattice alignment. These variations do not correlate with features in *S*(***k***), indicating a decoupling of static and dynamic correlations. This behavior persists down to near-particle length scales and reflects the persistence of long-lived, lattice-aligned collective modes supported by the toroidal topology of PBC.

In contrast, simulations performed under SBCs recover the expected static–dynamic correspondence. Both *S*(***k***) and *F*(***k***,*t*) depend only on | ***k*** |, and the extracted relaxation rates collapse onto a single isotropic curve consistent with de Gennes narrowing. Consequently, SBC recovers the scalar nature of collective relaxation rates expected for an isotropic bulk liquid on a finite computational domain.

### Local Structure and Microscopic Dynamics

The radial distribution function (Figure 5A) shows that the ghost interface successfully completes coordination shells up to the cutoff, maintaining the nodal structure of the liquid. The vertical shift in *g*(*r*) indicates a slight increase of the internal density in the core of the domain (∼5%) due to the effective surface tension of the radial boundary condition.

This thermodynamic perturbation does not affect microscopic dynamics: the statistics of interparticle collisions and cage-rattling time scales (Figure 5C,D) are indistinguishable from periodic benchmarks.

The fact that collisional statistics do not reflect the density inhomogeneity is expected. The vertical shift in *g*(*r*) reflects a weak redistribution of particles toward the interior. This perturbation is spatially smooth and acts on system scales. Local microscopic dynamics, however, are governed by short-range interactions and local coordination. Accordingly, the statistics of consecutive collisions (Figure 5D) are indistinguishable from periodic benchmarks. Rare-event observables such as per-pair waiting times sample the full domain and self-average over the weak density modulation. Together, these results confirm a clean separation of scales: SBC modifies global topology to correct hydrodynamic anisotropy without altering local collisional mechanisms.

**Figure 4:**
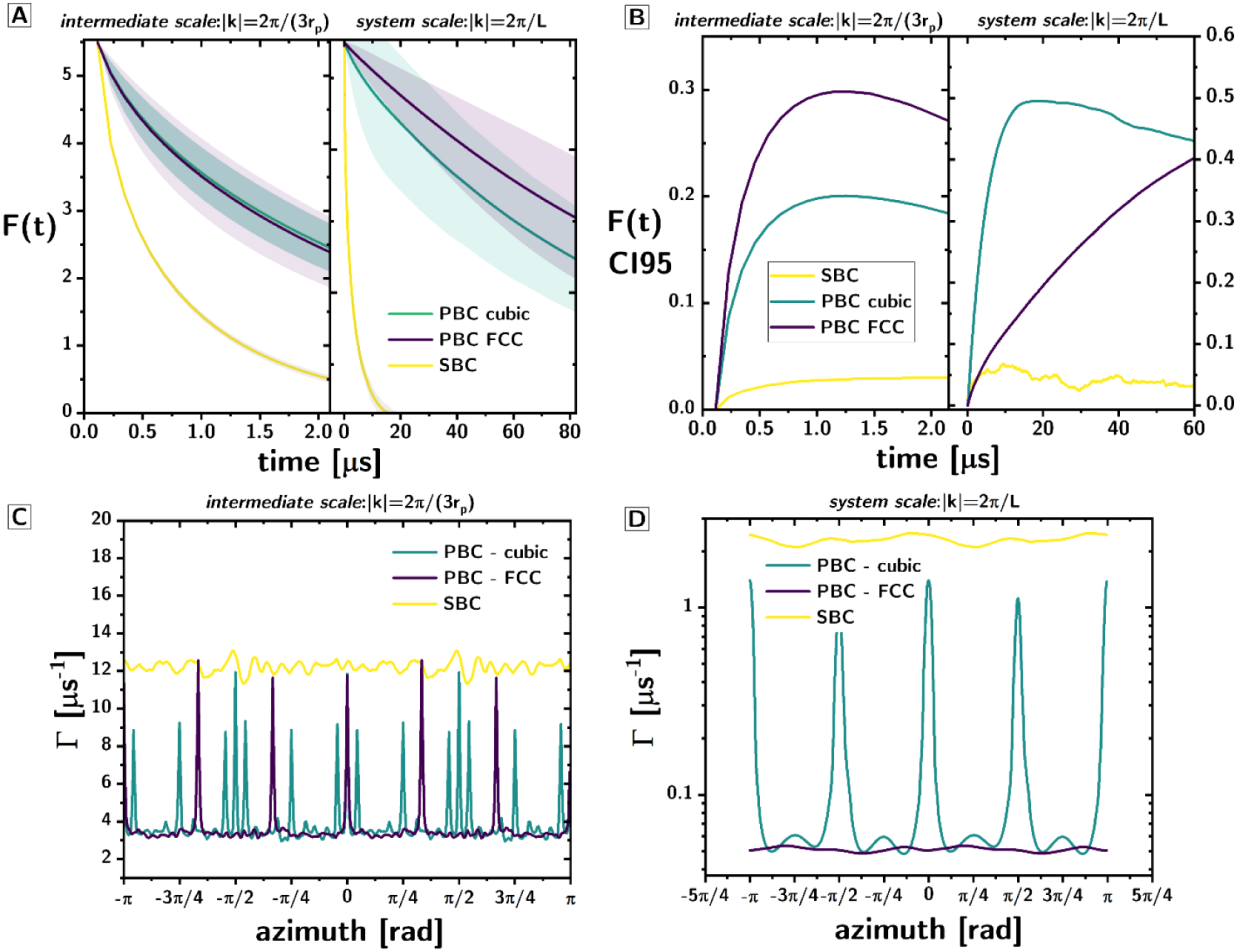
Angular dependence of relaxation rates under periodic and spherical boundary conditions. (A) Intermediate scattering function *F*(| ***k*** |,*t*) evaluated at two wavevector magnitudes: mesoscopic scale | ***k*** | ≅ 2π/3*r*_p_ (left) and system scale | ***k*** | ≅ 2π/*L* (right). Solid lines indicate the angular mean; shaded regions denote the 95% confidence interval across all directions. (B) Time evolution of the directional standard deviation σ_*F*(_ *_t_*_)_, quantifying the angular spread of the decay. (C, D) Azimuthal dependence of the relaxation rate Γ­(θ) extracted from the initial decay of *F*(***k***,*t*). At the mesoscopic scale (C), periodic boundaries (teal, purple) exhibit sharp peaks aligned with lattice vectors, indicating preferential relaxation channels, whereas SBC (yellow) is rotationally invariant. At the system scale (D), cubic PBC displays severe 4-fold anisotropy. The FCC signal is suppressed (flat, low magnitude) due to the absence of allowed reciprocal vectors in the scan plane (a spectral gap), while SBC maintains perfect isotropy with a globally faster rate reflecting the stiffness of the domain’s fundamental breathing mode.

### Symmetry and Conservation Laws

Periodic boundaries preserve linear momentum (translational invariance), but the coordinate-wrapping operation explicitly violates the conservation of angular momentum due to the algorithmically imposed teleportation. Mathematically, the wrapping of a particle across a box face acts as an instantaneous impulse torque applied to the system. Since the box is cubic, these impulses are not random; they are geometrically quantized along the lattice planes, injecting rotational noise that is strongly correlated with the Cartesian axes.

The SBC boundary couples the system to a rotationally isotropic environment. While the boundary does exchange angular momentum with the particles (via the unconstrained tangential degrees of freedom), it does so stochastically and uniformly across the solid angle. As shown in the Supporting Information (cf. Figure S2), this results in a diffusive, isotropic evolution of the system’s total angular momentum, avoiding the anisotropic shocks characteristic of the periodic lattice.

## Caveats and Limitations

### Space Filling

Unlike cubic domains, spheres cannot tile 3D space. SBC is therefore unsuitable for studying crystals or phases that require the indefinite periodic replication of a unit cell.

### Thermodynamic Compression

The tether rule imposes a decrease of the radial number density past the boundary. The drop in density causes an effective surface tension. As observed in *g*(*r*), this results in a redistribution of density as compared to a periodic box of equivalent volume.

### Lowest-Order Spherical Mode

The *l* = 0 spherical Bessel component, corresponding to purely radial density fluctuations, exhibits the strongest sensitivity to a spherical boundary topology. Unlike higher-order angular modes (*l* > 0), which possess tangential structure and efficiently phase-mix in the unfolded angular directions of SBC, the *l* = 0 mode lacks tangential degrees of freedom and therefore directly samples the global radial folding of the domain. Its deviation from higher-order modes in *S*(***k***) thus reflects enhanced sensitivity to finite-size topology rather than an effective stiffening or dissipative boundary effect. The faster decay rates observed under SBC remain attributable to the absence of coherent mode recurrence, not to modified local dynamics.

### Applicability to Newtonian Molecular Dynamics (MD)

Because SBC operates strictly as a kinematic exchange at the domain boundary, it does not alter the underlying equations of motion in the bulk. Consequently, it inherently preserves hydrodynamic interactions. While the results presented here employ overdamped BD, where the solvent acts as an isotropic momentum sink, the SBC framework is fully and directly applicable to standard Newtonian Molecular Dynamics (MD). In Newtonian Molecular Dynamics (MD), where momentum is locally conserved, the “channeling” effects of the periodic lattice are expected to be stronger due to the absence of frictional damping and the resulting persistence of long-wavelength hydrodynamic modes. Thus, the anisotropy reported here likely represents a lower bound on the artifacts present in standard atomic MD.^41^

**Figure 5:**
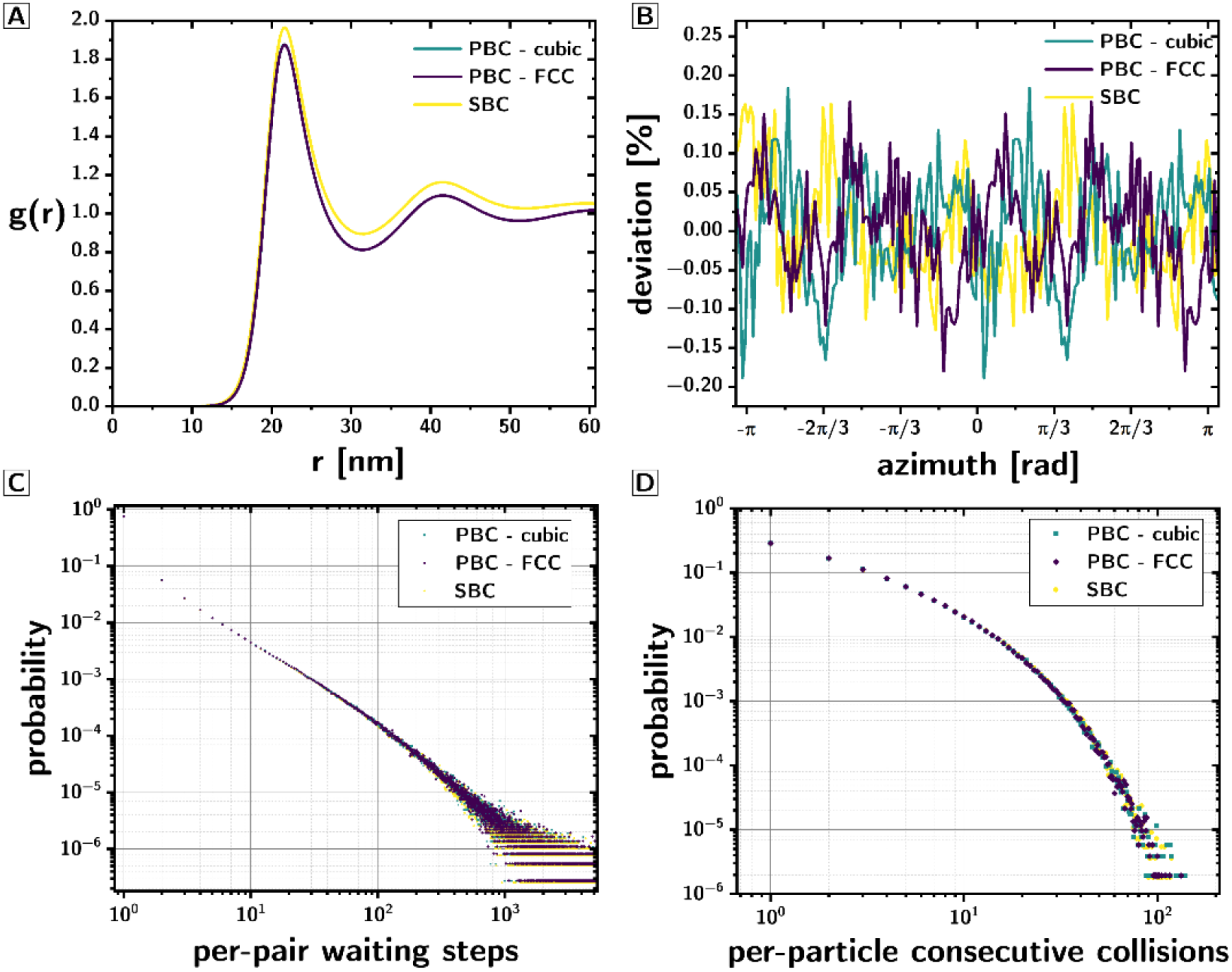
Local structure and microscopic dynamics under periodic and spherical boundary conditions. Comparison of short-range observables for dense LJ liquids under Spherical (SBC, yellow) and Periodic (cubic, teal, and FCC, purple) boundary conditions. (A) Radial distribution function *g*(*r*) plotted up to the interaction cutoff. SBC preserves the characteristic liquid structure (peak positions and widths) of the periodic bulk. The slight elevation in amplitude reflects a minor increase in effective core density (∼5%) induced by the surface tension of the radial boundary constraint. (B) Deviation of the angular neighbor distribution in the first coordination shell. Fluctuations are comparable to PBCs (<0.2%) and random, confirming that SBC maintains local structural isotropy. (C, D) Probability distributions of per-pair waiting times and per-particle consecutive collision counts. The distributions for SBC collapse onto the periodic benchmarks, demonstrating that the boundary modification does not perturb the local collisional mechanisms governing cage dynamics.

## Conclusions

We have demonstrated that the choice of boundary condition dictates the symmetry of collective dynamics in finite simulations. Periodic Boundary Conditions, while effective for scalar thermodynamics, impose a lattice topology that fractures the hydrodynamic propagator into discrete allowed channels. This results in severe directional anisotropy in transport coefficients that persists from the hydrodynamic limit down to mesoscopic length scales.

Spherical Boundary Conditions resolve anisotropy in collective dynamics with a flux-balanced domain whose topology is radially identified but tangentially open. By enforcing radial continuity while leaving tangential degrees of freedom unconstrained, SBC recovers the rotational symmetry of the bulk fluid. We hope SBC could be useful as a diagnostic and reference tool for assessing boundary-induced anisotropy in simulations of dense fluids and soft matter systems.

## Supplementary Material



## Notes

Demonstration of the effectiveness of spherical “masking” in recovering isotropic *S*(***k***) in PBCs. Characterization of the angular momentum temporal variations in the three boundary conditions analyzed in the manuscript (PDF)

## References

[1] Hansen, J.-P. ; McDonald, I. R. Theory of simple liquids: with applications to soft matter; Academic press, 2013.

[2] Boon, J. P. ; Yip, S. Molecular hydrodynamics; Courier Corporation, 1991.

[3] Dhont, J. K. G. An introduction to dynamics of colloids; Elsevier, 1996; Vol. 2.

[4] Berthier L., Biroli G. (2011). Theoretical perspective on the glass transition and
amorphous materials. Rev. Mod. Phys..

[5] Zaccone A., Dorsaz N., Piazza F., De Michele C., Morbidelli M., Foffi G. (2011). Crowding, Intermolecular Interactions,
and Shear Flow Effects in the Diffusion Model of Chemical Reactions. J. Phys. Chem. B.

[6] Lappala A., Zaccone A., Terentjev E. M. (2013). Ratcheted Diffusion Transport through
Crowded Nanochannels. Sci. Rep..

[7] Zaccone A., Terentjev E. M. (2012). Theory of molecular crowding in Brownian hard-sphere
liquids. Phys. Rev. E.

[8] Zaccone A., Terentjev E. M. (2012). Theory
of Thermally Activated Ionization and Dissociation
of Bound States. Phys. Rev. Lett..

[9] Zaccone A. (2013). Note: Slowing-down
of diffusion-controlled reactions in dense liquid matter. J. Chem. Phys..

[10] Allen, M. P. ; Tildesley, D. J. Computer Simulations of Liquids; Oxford University Press: New York, 1991.

[11] Frenkel, D. ; Smit, B. Understanding molecular simulation: From algorithms to applications; Elsevier, 2023.

[12] Privman, V. Finite size scaling and numerical simulation of statistical systems; World Scientific, 1990.

[13] Dünweg, B. ; Ladd, A. J. Lattice Boltzmann simulations of soft matter systems Advanced Computer Simulation Approaches For Soft Matter Sciences III Springer 2009 89–166 10.1007/978-3-540-87706-6_2

[14] Reible B. M., Hille J. F., Hartmann C., Delle Site L. (2023). Finite-size
effects and thermodynamic accuracy in many-particle systems. Phys. Rev. Res..

[15] Heidari M., Kremer K., Potestio R., Cortes-Huerto R. (2018). Fluctuations,
Finite-Size Effects and the Thermodynamic Limit in Computer Simulations:
Revisiting the Spatial Block Analysis Method. Entropy.

[16] Leocmach M., Tanaka H. (2012). Roles of icosahedral
and crystal-like order in the
hard spheres glass transition. Nat. Commun..

[17] Yeh I.-C., Hummer G. (2004). System-size dependence of diffusion coefficients and
viscosities from molecular dynamics simulations with periodic boundary
conditions. J. Phys. Chem. B.

[18] Hasimoto H. (1959). On the periodic
fundamental solutions of the Stokes equations and their application
to viscous flow past a cubic array of spheres. J. Fluid Mech..

[19] Salacuse J., Denton A., Egelstaff P. (1996). Finite-size
effects in molecular
dynamics simulations: Static structure factor and compressibility.
I. Theoretical method. Phys. Rev. E.

[20] Tuckerman, M. E. Statistical mechanics: Theory and molecular simulation; Oxford university press, 2023.

[21] Dünweg B., Kremer K. (1993). Molecular dynamics simulation of a polymer chain in
solution. J. Chem. Phys..

[22] Chandler, D. Introduction to modern statistical Mechanics; Oxford University Press: UK, Oxford, 1987.

[23] Binder, K. Finite size effects at phase transitions. In Computational Methods in Field Theory: Proceedings of the 31. Internationale Universitätswochen für Kern-und Teilchenphysik Schladming; Springer: Austria, 2005; pp. 59–125.

[24] Delville A., Pellenq R. J.-M., Caillol J. M. (1997). A Monte
Carlo (N,V,T) study of the
stability of charged interfaces: A simulation on a hypersphere. J. Chem. Phys..

[25] Wedekind J., Reguera D., Strey R. (2006). Finite-size effects in simulations
of nucleation. J. Chem. Phys..

[26] Chowdhury D., Schadschneider A., Nishinari K. (2024). Physics of collective transport and
traffic phenomena in biology: Progress in 20 years. Phys. Life Rev..

[27] Höfling F., Franosch T. (2013). Anomalous transport in the crowded
world of biological
cells. Rep. Prog. Phys..

[28] Brooks C., Karplus M. (1983). Deformable
stochastic boundaries
in molecular dynamics. J. Chem. Phys..

[29] Praprotnik M., Site L. D., Kremer K. (2008). Multiscale
simulation of soft matter:
From scale bridging to adaptive resolution. Annu. Rev. Phys. Chem..

[30] Cagin T., Pettitt B. M. (1991). Grand molecular
dynamics: A method for open systems. Mol. Simul..

[31] Toxvaerd S. (1981). The structure
and thermodynamics of a solid–fluid interface. J. Chem. Phys..

[32] Snook I., van Megen W. (1979). Structure
of dense liquids at solid interfaces. J. Chem.
Phys..

[33] Delgado-Buscalioni R., Coveney P. (2003). USHER: an algorithm for particle insertion in dense
fluids. J. Chem. Phys..

[34] Crank, J. The mathematics of diffusion; Oxford university press, 1979.

[35] Im W., Berneche S., Roux B. (2001). Generalized solvent boundary potential
for computer simulations. J. Chem. Phys..

[36] Beglov D., Roux B. (1994). Finite representation
of an infinite bulk system: Solvent boundary
potential for computer simulations. J. Chem.
Phys..

[37] Caillol, J.-M. A symplectic integrator for molecular dynamics on a hypersphere. arXiv, 2020, 10.48550/arXiv.2003.02600.

[38] Caillol J. M. (1993). A new potential
for the numerical simulations of electrolyte solutions on a hypersphere. J. Chem. Phys..

[39] Nissfolk J., Ekholm T., Elvingson C. (2003). Brownian dynamics
simulations on
a hypersphere in 4-space. J. Chem. Phys..

[40] Råsmark P. J., Ekholm T., Elvingson C. (2005). Computer simulations of polymer chain
structure and dynamics on a hypersphere in four-space. J. Chem. Phys..

[41] Alder B. J., Wainwright T. (1970). Decay of the
velocity autocorrelation function. Phys. Rev.
A.

