# Supplementary material for: Spherical Boundary Conditions: A Topological Framework for Isotropic Collective Dynamics
Source: J Phys Chem B. 2026 May 28;130(26):6660–9. doi: 10.1021/acs.jpcb.6c01770 (PMC13339638; doi:10.1021/acs.jpcb.6c01770)
Supplement: Supplementary file 1 [file jp6c01770_si_001.pdf]

SPHERICAL BOUNDARY CONDITIONS: A TOPOLOGICAL FRAMEWORK FOR ISOTROPIC COLLECTIVE DYNAMICS

MANUEL DEDOLA, LUDOVICO CADEMARTIRI[*]

Department of Chemistry, Life Sciences and Environmental Sustainability, University of Parma, Parco Area delle Scienze 17 A, Parma, Italy

[*] *Author to whom correspondence should be addressed: ludovico.cademartiri@unipr.it*

SUPPORTING INFORMATION

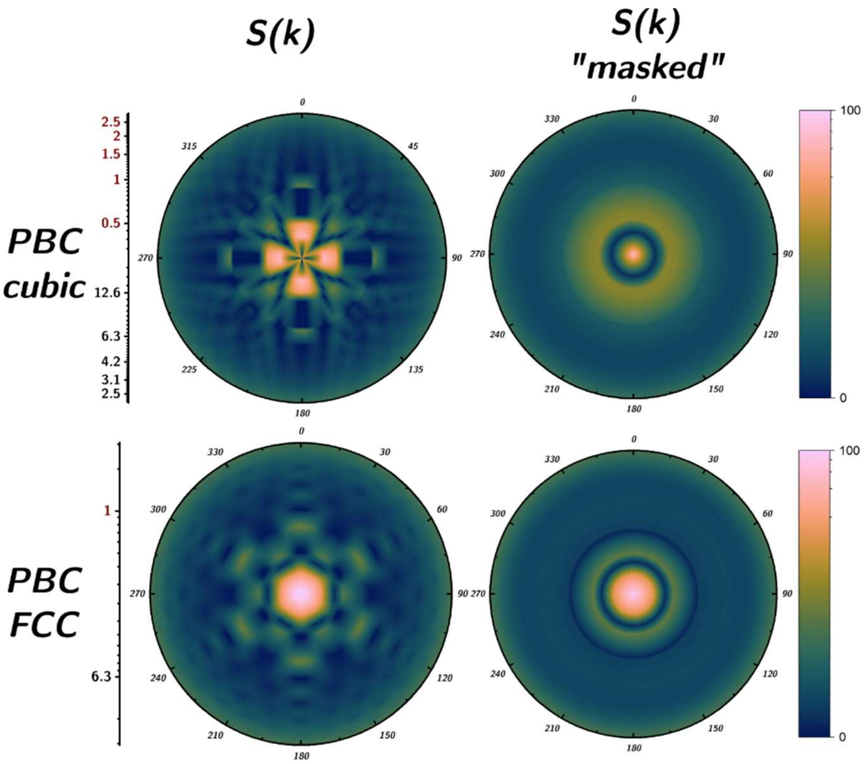

Figure S1. Static structure factor (N=1000, LJ potential, particle radius 10nm, $\varepsilon = 1$ k_BT, interaction cutoff = 3σ, $\Delta t = 200\tau_r$ (inertial relaxation time), integrated timesteps = 10^6) calculated from PBC conditions in a cubic and primitive FCC cell (left panels). The right panels are the same calculation performed by considering only particles within the largest sphere inscribed in the domain.

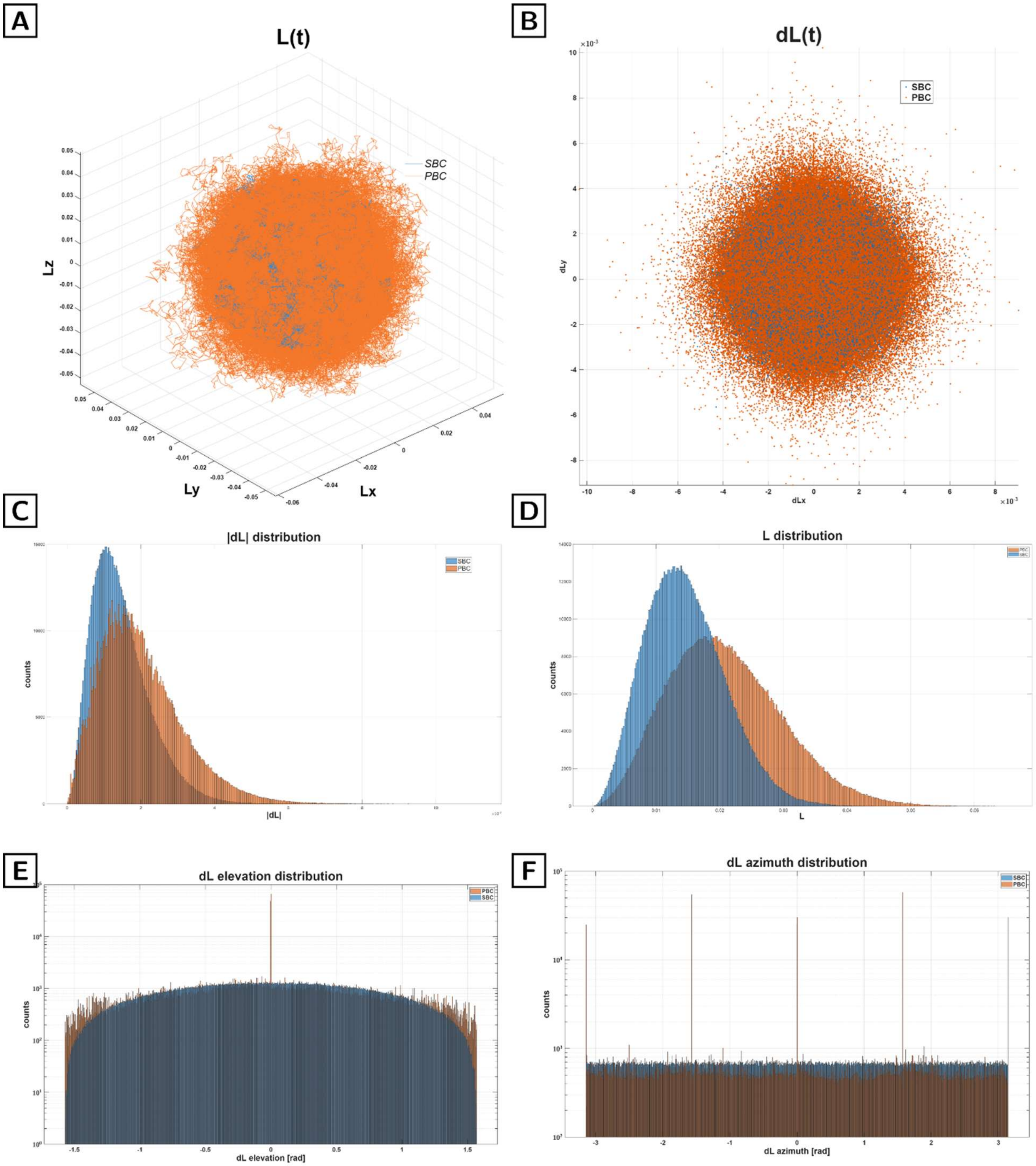

Figure S2. Angular momentum characterization. An ad hoc ballistic simulation was conducted to test the effect of the boundary conditions on the total angular momentum **L** ($N=10^3$, timesteps$=10^6$, $std_x=10^{-3}$, $r_p=10^{-2}$, $R=0.5$, MB distribution of velocities). We compared PBCs with SBCs. SBC implementation scrambled exiting particles by injecting a new real particle at a random position at the boundary with random velocity vector extracted from a MB distribution. In all panels, SBC is blue, PBC is orange. **(A)** trajectory of **L** in time. **(B)** **dL**(t)=**L**(t)-**L**(t-1): anisotropicity of dL "kicks" is evident in PBC.

(C) distribution of |**dL**|: notice the difference in median, width and smoothness. **(D)** distribution of |**L**|: notice the difference in median and width. **(E)** distribution of the elevation angle (angle above or below the xy plane, ∈ [-π/2, π/2]) in semilog scale: notice the huge spike at 0 in PBC. (F) distribution of the azimuth angle (angle in the xy plane, from x axis, ∈ [-π, π]) in semilog scale: notice the huge spikes at nπ/2 in PBC.

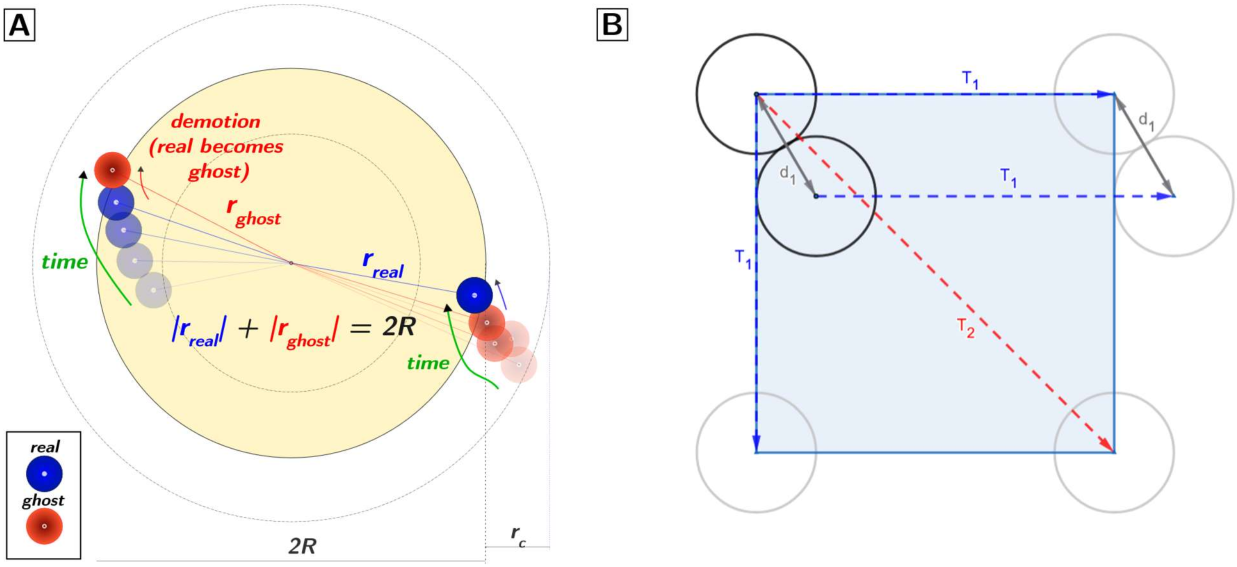

Figure S3. Comparison between SBC and PBC. (A) Schematic of the spherical boundary condition (SBC), illustrating the relation between real and ghost particles and the interaction halo of thickness r_c. **(B)** Schematic of periodic boundary conditions (PBC), illustrating the relation between a real particle (black solid circle) and its periodic images (ghosts, faded circles). As the real particle moves a distance d_1 inside the cell, all ghost particles are displaced by the exact same distance d_1 due to the rigid translational vectors along the edges (T_1) and the diagonal (T_2). For convenience, only the central unit cell is shown.

Radial Pair Distribution Function, g(r)

we computed g(**r**) by taking the ratio between the distance histograms of the simulated system and an ideal gas reference:

$$g(\boldsymbol{r}) = \frac{H_{sim}(\boldsymbol{r})}{H_{ig}(\boldsymbol{r})}$$

where $H_{sim}(\boldsymbol{r})$ is the time-averaged histogram of pairwise distances extracted from the thermalized simulation, and $H_{ig}(\boldsymbol{r})$ is the reference histogram obtained via Monte Carlo sampling of non-interacting ideal gas particles randomly distributed within the exact same bounding geometry.

Static Structure Factor, S(k)

The discrete Fourier sum was computed directly by projecting the exact instantaneous particle coordinates $\mathbf{r}_j$ onto the selected reciprocal space wavevectors $\mathbf{k}$:

$$S(\boldsymbol{k}) = \frac{1}{N}\left\langle \left| \sum_{j=1}^{N} e^{-i\boldsymbol{k}\boldsymbol{r}_j} \right|^2 \right\rangle$$

where N is the total number of particles and $\langle \ldots \rangle$ denotes the ensemble average evaluated over independent configurational snapshots sampled from the thermalized steady-state trajectory.

Intermediate Scattering Function, F(k,t)

To characterize the collective relaxation dynamics, we computed the intermediate scattering function, representing the time autocorrelation of the Fourier density components:

$$F(\boldsymbol{k}, t) = \left\langle \frac{1}{N} \sum_{j=1}^{N} \sum_{l=1}^{N} e^{-i\boldsymbol{k}\left(\boldsymbol{r}_j(t) - \boldsymbol{r}_l(0)\right)} \right\rangle$$
